# Bevacizumab exacerbates sinusoidal obstruction syndrome (SOS) in the animal model and increases MMP 9 production

**DOI:** 10.18632/oncotarget.25021

**Published:** 2018-04-24

**Authors:** Azin Jafari, Hanno Matthaei, Sven Wehner, Tolga Tonguc, Jörg C. Kalff, Steffen Manekeller

**Affiliations:** ^1^ Department of Surgery, University Hospital Bonn, Germany

**Keywords:** SOS, sinusoidal obstruction syndrome, bevacizumab, colorectal cancer, CRC

## Abstract

**Background:**

Thanks to modern multimodal treatment the ouctome of patients with colorectal cancer has experienced significant improvements. As a downside, agent specific side effects have been observed such as sinusoidal obstruction syndrome (SOS) after oxaliplatin chemotherapy (OX). Bevazicumab targeting VEGF is nowadays comprehensively used in combination protocols with OX but its impact on hepatotoxicity is thus far elusive and focus of the present study.

**Results:**

After MCT administration 67% of animals developed SOS. GOT serum concentration significantly increased in animals developing SOS (*p* < 0.001). Subsequent to MCT administration 100% of animals treated with Anti-VEGF developed SOS. In contrast, animals receiving VEGF developed SOS merely in 40% while increasing the VEGF dose led to a further decrease in SOS development to 25%. MMP 9 concentration in animals developing SOS was significantly higher compared to controls (*p* < 0,001). Additional treatment with Anti-VEGF increased the MMP 9 concentration significantly (*p* < 0,05).

**Conclusions:**

Preservation of liver function is a central goal in both curative and palliative treatment phases of patients with CRC. Thus, knowledge about hepatotoxic side effects of chemotherapeutic and biological agents is crucial. From the results it can be concluded that Anti-VEGF exacerbates SOS paralleled by MMP 9 production. Therefore, OX-Bevacizumab combination therapies should be administered with caution, especially if liver parenchyma damage is apparent.

**Methods:**

Male Sprague-Dawley rats were gavaged Monocrotaline (MCT) to induce SOS. Recombinant VEGF or an Anti-VEGF antibody was administered to MCT-treated rats and the hepatotoxic effect monitored in defined time intervals. MMP 9 expression in the liver was measured by ELISA.

## INTRODUCTION

The outcome of patients with colorectal cancer (CRC) has improved considerably over the past decades. Nevertheless, CRC represents the second most frequent malignant tumor and is responsible for over 200.000 deaths in Europe *per annum* [1]. The most important prognostic factor for the overall survival is the existence of metastases occuring in 50% of patients. Owing to the predominent blood drainage of the colorectum into the mesentericoportal venous system the liver presents as the most commonly affected organ by distant spread pushing the liver into the centre of interdisciplinary treatment efforts [2, 3].

Since its European approval in 1996 Oxaliplatin (OX), a DNA synthesis inhibitor, is one of the most important backbones in the battle against CRC. In spite of its proven antitumoral capabilities there are several relevant side effects associated with this drug. The more common ones include gastrointestinal symptoms such as nausea and diarrhea, neuropathy, changes to the blood count with associated infection and/or hemorrhagic disorders. [4, 5]. SOS of the liver, formerly known as hepatic veno-occlusive disease [6–8], is another latent and far less appreciated but severe drug-induced side effect. It has to be stressed that it is remarkably common with a prevalance of up to 60% subsequent to OX administration [6, 9, 10].

Morphologically, SOS associated damage to the liver parenchyma starts with rounding up and swelling of sinusoidal endothelial cells leading to a destruction of their characteristic lining architecture. This is followed by sinusoidal occlusion resulting in hemorrhagic necrosis of larger areas of liver parenchyma eventually leading to hepatic functional insufficiency [6–8, 11, 12]. The sometimes delayed course of SOS is not simply a problem of impaired liver function *per se*. This chronic state may also severely complicate liver directed ablative therapies (i.e. RFA) or surgical resection thereby compromising the individual oncologic treatment plan resulting in a decreased long-term survival. To date, no protective treatment has been established with regard to SOS development [8, 13–16]. Bevacizumab, increasingly used in combination regiments with OX is a humanized monoclonal antibody targeting vascular endothelial growth factor-A (VEGF-A). This potent antiangiogenetic drug is among the most promising biologicals in the treatment of advanced CRC to date [17–19]. Interestingly, several studies reported a hepato-protective effect of Bevacizumab with respect to development and progression of SOS without explaining a possible pathomechanism [20–25]. Other investigations reported its altogether negative hepatic impact particularly compromising liver regeneration [26–28]. Thus, the current evidence level regarding this topic is low and experimental data are limited making a conclusion about the effect of Bevacizumab on existing SOS impossible. However, since regenerative capabilities of hepatic parenchyma are essential the issue of drug hepatotoxicity is a central element in modern interdisciplinary approach and specific answers are substantially required. Hence, in the present study the impact of Bevacizumab on SOS was examined, for the first time, in a standardized experimental model of monocrotaline(MCT)- induced SOS *in vivo* to investigate the potential influence of an anti-angiogenetic agent on preexisting liver damage.

## RESULTS

### Group PG 1 and 2

**Figure 1:**
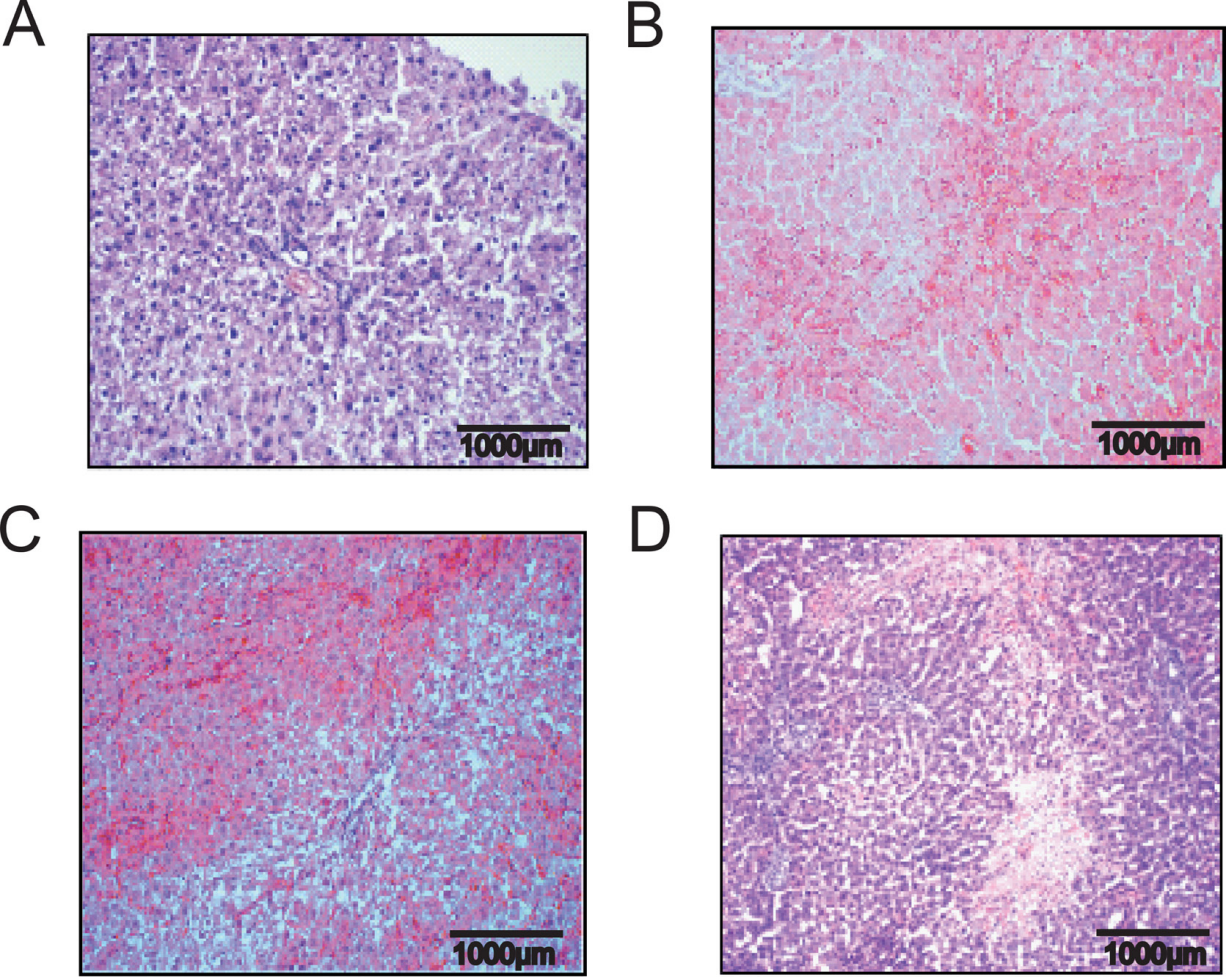
Hematoxylin and eosin staining liver tissue demonstrating time dependent SOS development Results from group PG 1; Animals were treated with MCT and sacrificed at day 1, 2 or 4 and liver tissue and blood samples harvested. (**A**) control animal with no MCT treatment and healthy liver tissue; (**B**) beginning pathological changes at 24 h after treatment with 90 mg/kg/BW MCT; (**C**) characteristic signs of SOS at 48 h; (**D**) severe SOS at 96 h.

**Figure 2:**
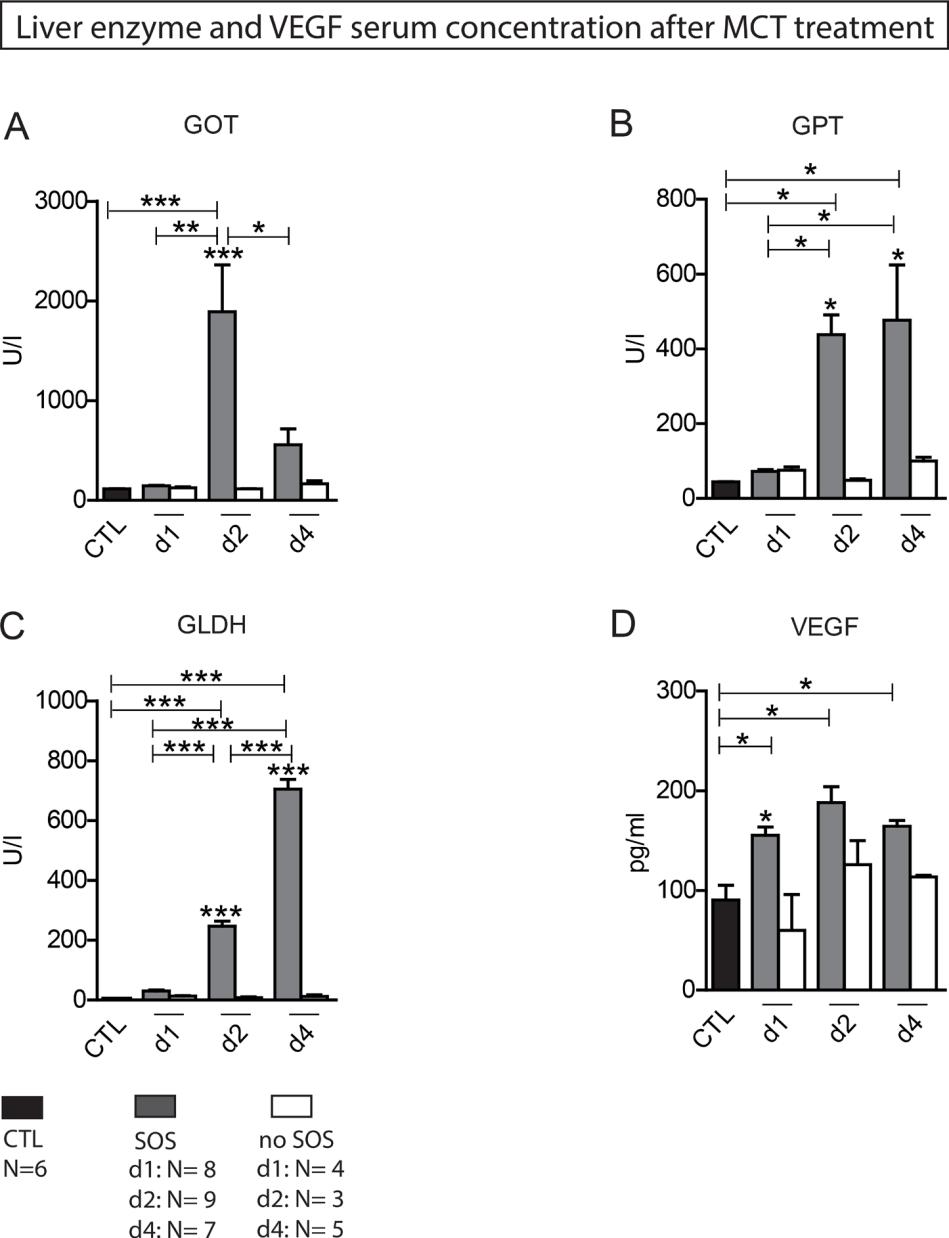
Liver enzyme and VEGF serum concentration after MCT treatment Results from group PG 1; Animals were treated with MCT and sacrificed at day 1, 2 or 4 and liver tissue and blood samples harvested. Histological examination of liver tissue determined wether or not SOS characteristics occurred. According to these results animals were divided in group SOS and group no SOS. Group CTL shows serum concentrations of untreated, healthy animals. (**A**) GOT serum concentration; (**B**) GPT serum concentration; (**C**) GLDH serum concentration; (**D**) VEGF serum concentration; Major significances are shown; ^*^p < 0,05; ^**^p < 0,005; ^***^p < 0,001 (CTL control group).

**Figure 3:**
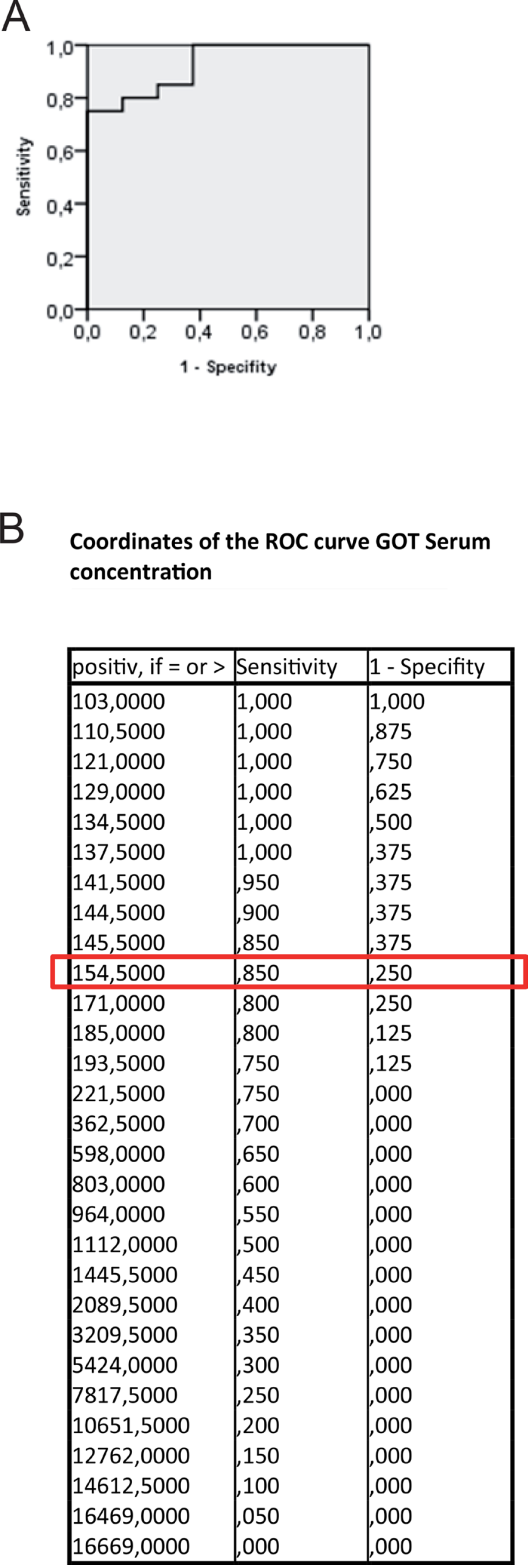
ROC curve for GOT serum concentration Results from group PG 1; ROC analysis were conducted; (**A**) shows the ROC curve; (**B**) shows possible cut-off values. At 154,5 U/l the highest possible sensitivity and specificity is given. The same procedure was performed for GPT, GLDH and VEGF concentrations. Results are summarized in Table 2.

In group PG1 animals showed either time-dependent histological signs of SOS or no changes at all (Figure 1A–1D). Overall 66.7% of animals treated with MCT showed respective histological alterations (24 h: *N* = 8; 48 h: *N* = 9; 96 h: *N* = 7). Minor morphological changes could already be observed after 24 h represented by a slightly more rounded outer shape of the cells compared to hepatocytes of untreated rats. At 96 h the histological characteristics of SOS were fully developed and corresponded to the extent of liver enzyme elevation (Table 1). Especially, the GOT levels were significantly elevated compared to animals with no histological changes after MCT administration. The peak concentration was seen on day two (Figure 2A). Similar results were obtained for GLDH and GPT serum concentrations, here levels remained elevated also on day four (Figure 2B–2C). Additionally, VEGF levels measured by ELISA showed a significant increase in animals with histomorphological SOS alterations starting after 24 h (Figure 2D). Through ROC curves, cut-off values with high sensitivity and specificity for the above mentioned parameters were calculated (Figure 3A–3B) that were implemented as prediction markers for SOS development in the following experiments (Table 2). The validity of calculated cutoffs was evidenced by results of PG2. A positive correlation between SOS prediction according to cutoff values for GPT, GOT, GLDH and VEGF (see method section) with final liver tissue examination could be shown in all animals (*N* = 12 of 12).

**Table 1:** Course of SOS development regarding enzyme levels and histology

	GOT	GPT	GLDH	VEGF	HE staining
Day 1	145 ± 12.96	72.25 ± 15.28	29.78 ± 7.881	163.1 ± 7.568	Beginning changes in sinosoids; mild erythrocyte congestion
Day 2	1890 ± 1152	438.3 ± 105	247.7 ± 37.02	188.1 ± 39.17	Destruction of characteristic sinusoidal lining structure; increased erythrocyte congestion
Day 4	557 ± 322.6	476.6 ± 330.6	704.9 ± 73.31	164.4 ± 10.4	Extensive damage to sinusoids; hemorragic necrosis

**Table 2:** Cut-off values

	GOT	GPT	GLDH	VEGF
Cut-off value	154.5 U/l	111 U/l	20.5 U/l	128,4 pg/ml
Sensitivity	85%	88.2%	95%	90.9%
Specificity	75%	88.9%	85.7%	75%

In summary, SOS development could be predicted by VEGF, GOT, GPT and GLDH serum levels 24 h after MCT ingestion.

### SOS treatment with Anti-VEGF

**Figure 4:**
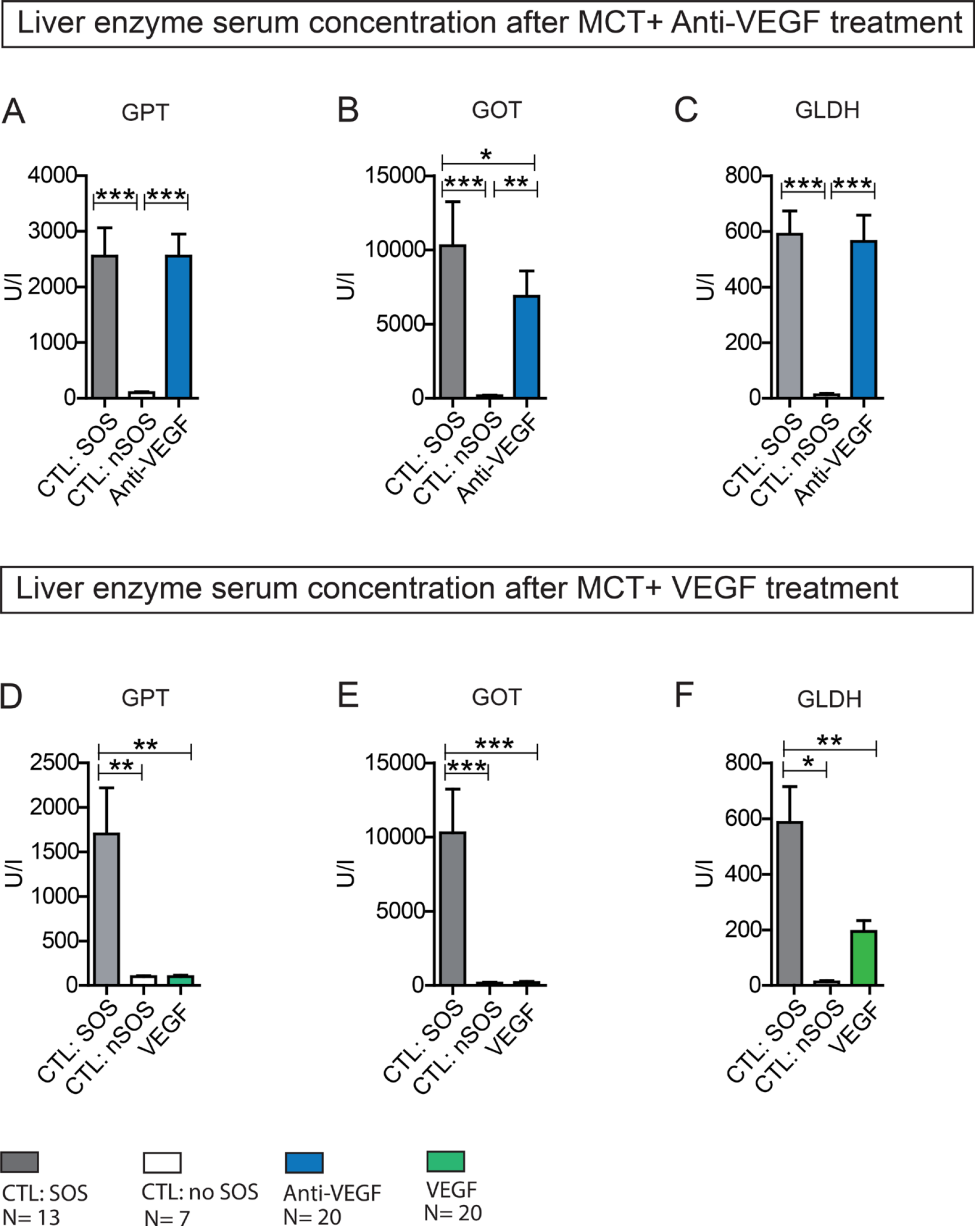
Liver enzyme serum concentration after MCT+ Anti-VEGF/VEGF treatment Results from group Anti-VEGF 1&2 plus VEGF 1&2; Animals were treated with MCT and additionally received Anti-VEGF (**A**–**C**) or VEGF (**D**–**F**). Blood samples and liver tissue were collected after 96h and examined. Results from group Anti-VEGF 1 and 2 are combined provided (A–C); Results from group VEGF 1 and 2 are combined provided; (A) GOT serum concentration after Anti-VEGF treatment; (B) GPT serum concentration after Anti-VEGF treatment; (C) GLDH serum concentration after Anti-VEGF treatment; (D) GOT serum concentration after VEGF treatment; (E) GPT serum concentration after VEGF treatment; (F) GLDH serum concentration after VEGF treatment; Major significances are shown; ^*^p < 0,05; ^**^p < 0,005; ^***^p < 0,001 (CTL SOS: control animals treated with MCT and PBS developing SOS; CTL: nSOS: control animals treated with MCT and PBS developing no SOS; Anti-VEGF: animals treated with MCT and Anti-VEGF; VEGF: animals treated with MCT and VEGF).

In Group Anti-VEGF 1 the blood samples taken 24 h after MCT treatment and prior to Anti-VEGF application indicated SOS development in 70% of animals. At 96 h after MCT administration all animals (*N* = 10) treated with Anti-VEGF developed SOS compared to 70% in the control group with PBS treatment. Thus, the induction rate of MCT for SOS was increased from 70 to 100% by the application of Anti-VEGF antibody. Similarly, in group Anti-VEGF 2 all animals with Anti-VEGF treatment developed an SOS at 96 h. As no difference regarding the time point of application (0 h vs. 24 h) was seen the results are combined in Figure 4A–4C. The histological findings were paralleled by significantly increased liver enzyme levels.

In summary, Anti-VEGF administration appeared to exacerbate SOS development.

### SOS treatment with VEGF

In Group VEGF 1 SOS development was predicted in 60% of animals according to the cut-off values. Animals treated with 750 ng/g/BW VEGF after MCT gavage showed only in 40% histological characteristics of SOS, compared to 70% in the control group with PBS administration. Thus, SOS induction was decreased from 60% to 40%. To investigate dosage-response relationship and a possible more profound effect group VEGF 1 and 2 were repeated with 1.5µg/g/BW VEGF. Increasing the dose led to a further decrease in SOS development down to 25% (0 h: *N* = 2; 24 h: *N* = 3). Once again, no differences regarding the time point of application (0 h vs. 24 h) were seen, therefore results are combined in Figure 4D–4F. In concordance with the histological results, liver enzyme levels were not elevated. The concentrations were similar to animals with only MCT treatment and no SOS development.

In summary, VEGF administration was observed to attenuate SOS development.

### MMP 9 levels after Anti-VEGF treatment

As an early increase of MMP 9 concentration during the course of SOS has been suggested as a possible pathogenetic trigger [29] MMP 9 production was examined by ELISA.

**Figure 5:**
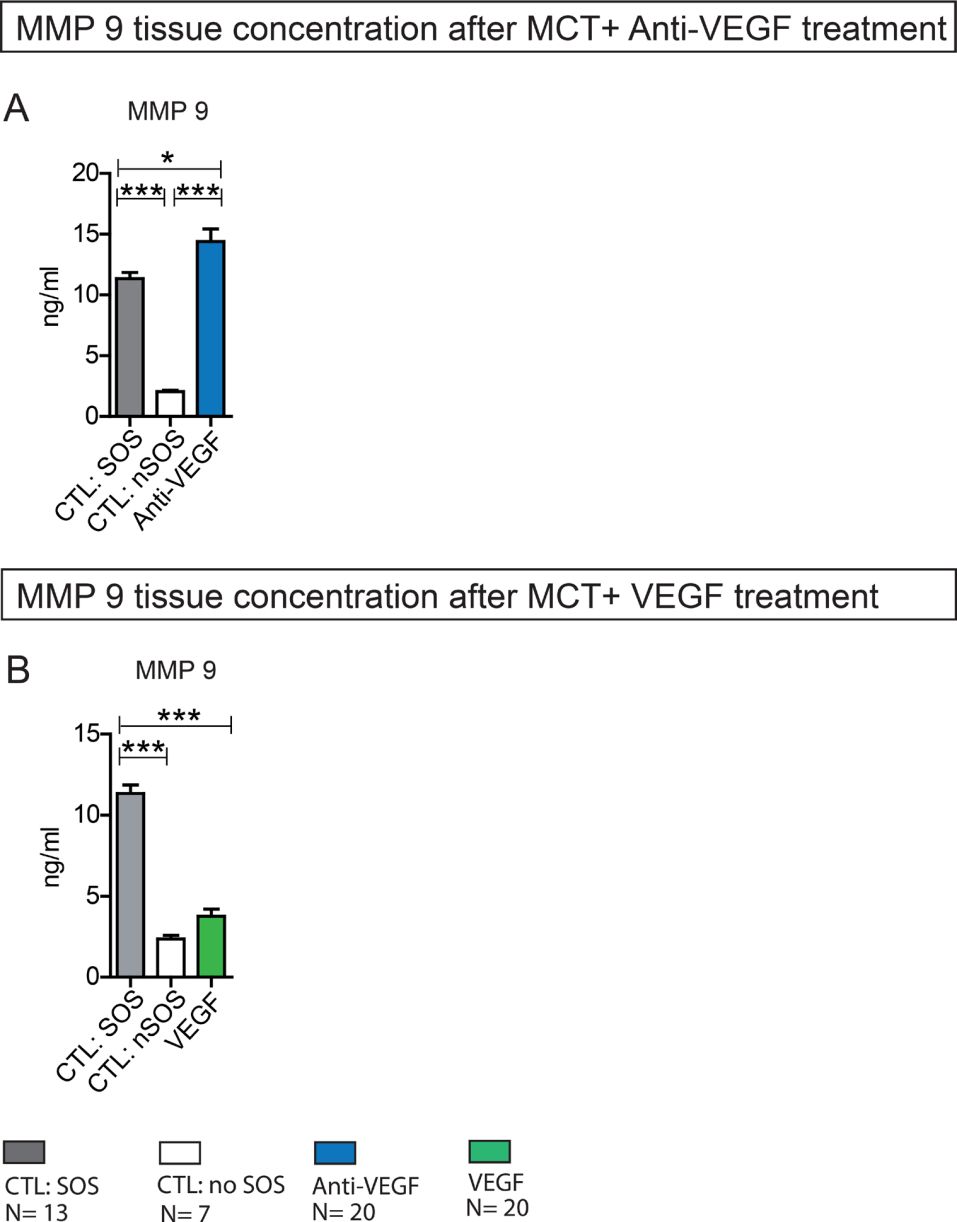
MMP 9 liver tissue concentration after MCT+ Anti-VEGF/VEGF treatment Results from group Anti-VEGF 1&2 plus VEGF 1&2; Animals were treated with MCT and additionally received Anti-VEGF or VEGF. Blood samples and liver tissue were collected after 96h and examined. Results from group Anti-VEGF 1 and 2 are combined provided (**A**); Results from group VEGF 1 and 2 are combined provided (**B**); Major significances are shown; ^*^p < 0,05; ^**^p < 0,005; ^***^p < 0,001 (CTL SOS: control animals treated with MCT and PBS developing SOS; CTL nSOS: control animals treated with MCT and PBS developing no SOS; Anti-VEGF: animals treated with MCT and Anti-VEGF; VEGF: animals treated with MCT and VEGF).

Accordingly, animals with MCT treatment and SOS development showed significantly higher MMP 9 tissue concentrations compared to animals with no SOS characteristics after MCT exposure. The additional application of Anti-VEGF further increased MMP 9 concentration significantly (Figure 5A).

In summary, Anti-VEGF appears to stimulate MMP 9 production.

### MMP 9 levels after VEGF treatment

In line with the above described results, the application of VEGF resulting in low SOS development rates did not influence MMP 9 production. MMP 9 levels in VEGF-treated animals did not differ from animals with no SOS after MCT treatment alone (Figure 5B).

In summary, VEGF does not seem to influence MMP 9 production.

### Proliferation in SOS and the influence of Anti-VEGF/VEGF treatment

**Figure 6:**
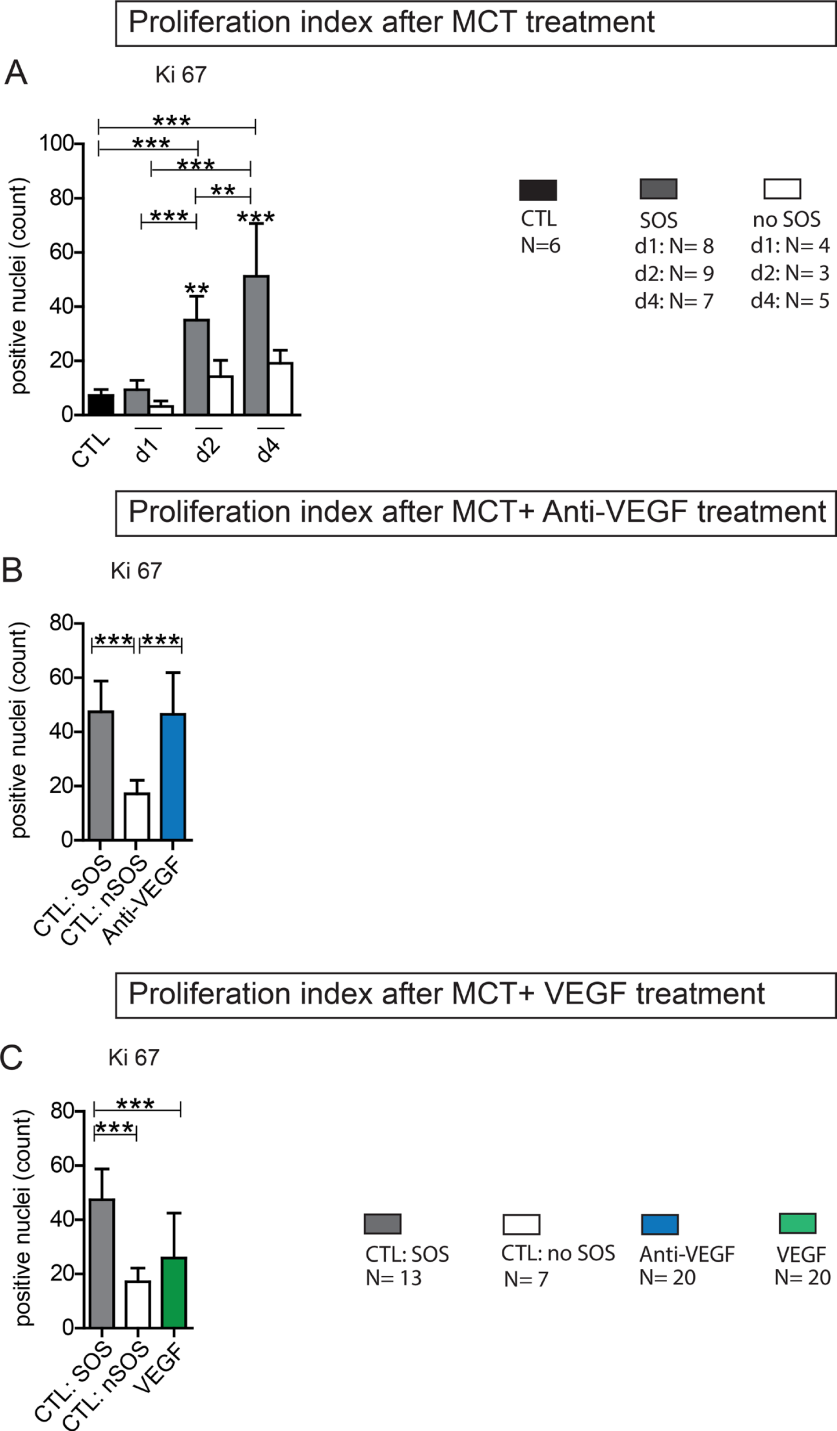
Ki 67 Proliferation index Ki 67 staining on liver tissue was performed and the positive cells were counted (from each section 3 perivenular and three periportal areas with 92332 µm^2^); (**A**) Results from group PG 1; Animals were treated with MCT and sacrificed at day 1, 2 or 4. Histological examination of liver tissue determined wether or not SOS characteristics occurred. According to these results animals were divided in group SOS and no SOS (CTL control group); (**B**) Results from group Anti-VEGF 1 and 2; Animals were treated with MCT and additionally received either simultaneously or after 24 h Anti-VEGF. Liver tissue was collected after 96 h. (**C**) Results from group VEGF 1 and 2; Animals were treated with MCT and additionally received either simultaneously or after 24 h VEGF; Major significances are shown; ^*^p < 0,05; ^**^p < 0,005; ^***^p < 0,001.

Animals with SOS exhibited significantly higher rates of Ki-67 positive cells compared to animals that failed to develop SOS (Figure 6A). Additional Anti-VEGF treatment resulted in proliferative indices comparable to non-treated SOS animals (Figure 6B). In accordance to the overall benefical effects regarding SOS VEGF application showed reduced levels of Ki-67^+^ cells, not differing from non-SOS animals (Figure 6B).

In summary, proliferation proved to be increased in SOS and is not inhibited by Anti-VEGF or stimulated by VEGF.

## DISCUSSION

Over the past decades multimodal cancer treatment has been on the rise. Paralelling this ongoing success of combination protocols including novel drugs such as antibody targeted therapies various known and yet unknown side effects are being carefully investigated. SOS, as a result of OX therapy, considerably impairs the outcome of patients with colorectal liver metastases with no preventive or therapeutic options [6–10]. The benefits of antiangiogenic Bevacizumab concerning tumor suppression have been clearly proven in several tumor entities [30]. While several adverse side effects of Bevacizumab such as hypertension, fatigue, diarrhea, a higher risk of hemorrhage and bowel perforation are well documented [31, 32] its possible hepatotoxicity alone or in combination with other drugs especially with regard to SOS is not entirely understood.

Thus far, such knowledge is merely based on retrospective studies analyzing the extent of SOS in patients after OX exposure with or without Bevacizumab [20–25]. For example, Rubbia Brandt *et al.* found SOS to be less frequent in patients with additional Bevacizumab treatment and this intriguing coherence was supported by Volk *et al.* in 2016 within a systematic review and meta-analysis [7, 25]. Still, controversial conclusions can be found in literature indicating a negative effect of Bevacizumab on SOS development [26, 28]. Due to heterogeneous and oftentimes small patient cohorts and differences in relevant study parameters such as duration and dose of treatment, underlying liver disease etc. these results must be interpreted with caution. Randomized controlled trials focussing on this issue are virtually missing and experimental data limited.

MCT administration is the standard for SOS induction within experimental animal studies [33]. However, as previously shown [34], MCT ingestion does not regularly result in histologically provable SOS formation. Therefore, we sought to establish a marker panel to predict SOS development. The elevated liver enzyme levels in MCT-induced SOS in our study are in line with the same enzymes measured in patients with OX-induced SOS showing the validity of our marker panel [6, 35]. In addition to liver enzyme levels also an increase of VEGF concentration has been shown to correlate with progressive SOS development in humans [36]. In the presented animal study, VEGF concentration similarly peaked 24 h after MCT administration proving again the validity of our model. As the elevation of liver enzyme levels in patients with colorectal liver metastases is rather unspecific for diagnosing SOS (e.g. due to concomitant cholangitis and necrosis), the determination of VEGF concentration in serum may be a more specific diagnostic tool. This, however, demands further investigation for a possible future clinical implementation. The study design in group PG 1 and 2 with individual treatment of respective animal cohorts and statistic computations was chosen in order to allow exclusion of possible coincidential effects and a subsequent precise evaluation of substances applied with respect to SOS. Ideally, the presented markers and their cutoffs can be used in future SOS research.

Surprisingly, investigating the influence of Anti-VEGF resulted in deterioration of SOS development. For further evidence the effect of VEGF itself was examined. Within the VEGF signal protein familiy VEGF-A is the the key player for angiogenesis, vascular permeability, stimulation of cell migration etc. [37], therefore recombinant rat VEGF-A was chosen for all experiments. In line with the results for Anti-VEGF application its counterpart VEGF attenuated SOS development impressively. The beneficial effects of VEGF in hepatic regeneration has been previously evidenced [27, 38] and could explain our findings. Besides its pro-angiogenic activity, VEGF stimulates cell migration in macrophage lineage, endothelial cells and enhances vascular permeability [37]. An uncertainty remained whether or not the positive influence of VEGF on SOS was due to its known general regenerative benefits on liver parenchyma or its potential interference in SOS pathomechanism. Therefore, and in order to clarify our findings regarding the negative influence of Anti-VEGF, we performed further investigations.

Till today the pathogenesis of SOS is not fully understood [33]. Deleve *et al.* discovered an early increase of MMP 9 concentration *in vitro* in MCT-treated liver cells [29]. MMP 9 belongs to the family of matrix metalloproteinases and impacts on extracellular matrix within various physiologic processes such as embryogenesis, wound healing, cell migration etc [39]. The investigators concluded that MCT leads to depolymerization of F-actin in sinusoidal endothelial cells resulting in an increased expression of MMP 9 and MMP 2. Similar results were reported by Hanumegowda *et al.* in an experimental animal model using MCT, showing a dose-dependent elevation of MMP 9 concentration, followed by loss of basement membrane collagen [40]. Hence, MMP 9 tissue concentrations were measured by ELISA in the presented study. An increase of MMP 9 concentration after MCT exposure paralleling histologic SOS development was observed. Interestingly, the application of Anti-VEGF enhanced MMP 9 production causing higher SOS induction rates. In contrast, VEGF application showed no influence on MMP 9 concentration and consequently SOS development was attenuated.

Furthermore, measurement of Ki-67 index excluded the afore known anti-proliferative side effects of Anti-VEGF as the reason for SOS exacerbation. Proliferation was not inhibited by additional application of Anti-VEGF.

To sum up, the results of the presented animal study suggest an increase of SOS development after Anti-VEGF treatment. As a potential proof of this pathomechanism an increase of MMP 9 concentration was observed. Additionally, VEGF attenuated SOS development and this finding could pave the way for future research on proangiogenic agents.

The suggested negative influence of Anti-VEGF with respect to SOS manifestation needs to be examined in humans before drawing final conclusions for clinical therapy. Hopefully, if confirmed in subsequent studies, VEGF-based therapies may be a treatment option for SOS. However, its administration will depend on the individual clinical setting (e.g. in case of metastasized tumor disease VEGF proved to be contraindicated).

## MATERIALS AND METHODS

### Animals

All animal experiments were performed in accordance with the federal German law regarding the protection of animals. The principles of laboratory animal care (NIH Publication No. 85–23, revised 1985) were followed. In all experiments male Sprague-Dawley rats, obtained from Charles River Laboratories, weighing 200–250 g, were used.

### Experimental design

#### SOS induction and prediction

**Figure 7:**
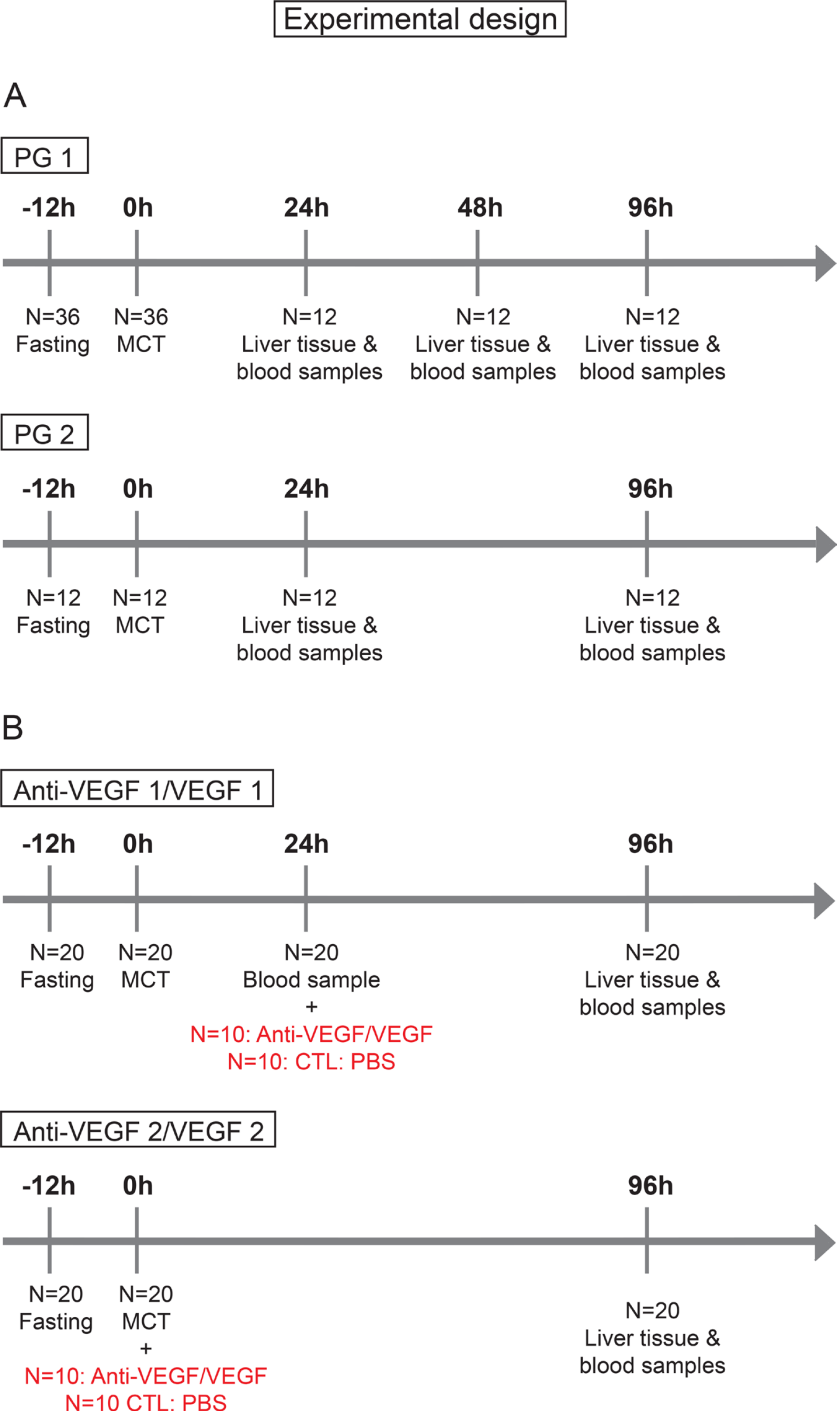
Timetable and experimental design (**A**) In group PG 1 N=36 animals were fasted for 12h prior to MCT gavage; at 24, 48 and 96 h *N* = 12 animals were sacrificed and liver tissue and blood samples harvested. In group PG 2: *N* = 12 animals were fasted for 12 h prior to MCT gavage; at 96 h *N* = 12 animals were sacrificed and liver tissue and blood samples harvested. (**B**) In group Anti-VEGF 1 *N* = 20 animals were fasted for 12 h prior to MCT gavage; 24 h after MCT treatment blood samples were taken from the tail vein and Anti-VEGF antibody administered to *N* = 10; the control group (CTL) *N* = 10 received PBS. In group VEGF 1 the same procedure was performed usind VEGF instead of Anti-VEGF. In group Anti-VEGF 2 the time point of antibody treatment was changed. Here, MCT and Anti-VEGF (*N* = 10) were applied simultaneously, the control group received PBS (*N* = 10). In group VEGF 2 the same procedure was performed usind VEGF instead of Anti-VEGF.

For SOS induction, as previously shown [34], animals were fasted for 12 h following the application of 90mg/kg/BW MCT (Sigma Aldrich, St. Louis, MO) by gavage under light isofluran anesthesia (Figure 7A). As MCT ingestion does not regularly result in SOS we stratified our animals into “Prediction Group 1 (PG1)” and “Prediction Group 2 (PG2)” to investigate, if SOS development can be predicted by serum analysis as outlined below. In PG 1 animals were sacrificed 24, 48 or 96 h after MCT gavage. For SOS examination liver tissue and blood samples from the inferior vena cava were taken immediately before sacrificing the animals. Subsequently, a correlation of the histologic pattern with respect to SOS and serum parameter levels was performed. For all serum parameters receiver operating characteristic (ROC) analyses were conducted and cut-off values set. The sensitivity and specificity of the cut-offs was validated by the results in PG2 experiments. Herein, blood was initially collected from the tail vein 24 h after MCT administration. According to the elevation of serum parameters SOS development was estimated. To validate the predictions, all animals were sacrificed at 96 h after MCT application and blood and liver samples were harvested followed by another correlation of prediction results and final histological findings.

### SOS treatment

SOS was induced as described above. In “Group Anti-VEGF 1” (Figure 7B) animals received 0.2 µg/g/BW recombinant rat Anti-VEGF (Fitzgerald Industries International, Acton, MA, USA) intravenously into the tail vein 24 h after MCT treatment. Prior to the application of Anti-VEGF blood samples were harvested from the tail vein for SOS prediction analyses. In “Group Anti-VEGF 2” (Figure 7B) the antibody was given simultaneously with MCT. 96 h after MCT application animals were sacrificed and blood samples and liver tissue harvested for further examinations. The application of recombinant rat VEGF (VEGF-A; R&DSystems, Minneapolis, MN, USA) was performed according to the same protocol referred to as “Group VEGF 1” and “Group VEGF 2”. Both groups were first performed with VEGF dose of 750 ng/g/BW and later on repeated with a higher dose of 1.5 µg/g/BW.

### Liver histology

Liver tissue was fixed in 4% paraformaldehyde, embedded in paraffin and sectioned at 4 µm. For SOS assessment the slides were stained with haematoxylin & eosin and Sirius red. The examination was performed blindly by a pathologist. SOS grading was determined based on changes regarding sinusoidal dilatation, nodular regeneration, centrilobular or portal vein lesions, centrilobular vein, and perisinusoidal fibrosis and steatosis. SOS was categorized as absent, mild, moderate or severe as outlined in our previous publication [34].

### Serum parameters

Activity of glutamic oxaloacetic and glutamate-pyruvate transaminase (GOT,GPT) and glutamate dehydrogenase (GLDH) served as serum markers for SOS related liver damage as previously shown [6, 34]. The measurements were performed spectrophotometrically using commercially available kits (Boehringer, Mannheim, Germany) according to standard laboratory techniques.

### Enzyme linked immuno absorbent assay (ELISA)

VEGF and matrix metalloproteinase (MMP) 9 concentration of serum and liver tissue samples were measured by ELISA according to the manufacturers’ instructions using Quantikine Rat VEGF and MMP 9 ELISA (R&DSystems, Minneapolis, MN, USA).

### Ki 67 immunostaining

Assessment of hepatocyte proliferation was performed by immunostaining for Ki 67. Briefly, liver sections (4µm) were deparaffinized and washed with PBS. After incubation with H2O2 for 15 minutes, the sections were rinsed in PBS and blocked for 30 minutes with goat-serum (Biozol, Eching Germany). The primary antibody Anti-Ki67 rabbit monoclonal antibody (ab 15580; Abcam, Cambridge, UK), diluted 1:300, was added, following an overnight incubation. Afterwards, a secondary antibody was applied (1:200, Rabbit-IgG, Biozol, Eching Germany) with 30 minutes incubation. After washing with PBS the sections were first incubated with ABC-solution (ABC-Kit, Biozol, Eching Germany) for 30 minutes followed by washing with PBS and finally rinsed in DAB solution for 2 minutes under observation. The reaction was stopped with distilled water. After counterstaining with haematoxylin the sections were evaluated under light microscopy. The positive cells were counted while from each section three perivenular and three periportal areas of 92332 µm^2^ were analyzed and the proliferation index generated .

### Statistics

Statistical analyses were perfomed using SPSS v. 20 (IBM) and Graph Pad Prism v. 5 (IBM). Data are expressed as means ± SD. Differences in the measured variables between each group were assessed using one-way Anova or two-way Anova. A *P* < 0,05 was considered to indicate significance.

## CONCLUSIONS

The presented study provides evidence to the pathway of SOS development under the influence of Anti-VEGF. Herein, MCT exposure induces SOS development which is accompanied by a serum liver transaminase increase and MMP 9 production. Anti-VEGF exacerbates SOS development and elevates liver transaminase levels and amplifies MMP 9 production. These results may have relevant clinical implications, especially with regard to the beneficial effect of VEGF as a potential cure to SOS. Patients with underlying liver disease or rapid elevation of liver enzyme levels after OX exposure may not be considered for additional Bevacizumab treatment. This may be particularly important for patients in whom liver directed therapies in a multimodal setting may further impair liver function why these *in vivo* data need further clinical validation.

## Abbreviations

OX: Oxaliplatin
SOS: sinusoidal obstruction syndrome
VEGF: vascular endothelial growth factor
MCT: Monocrotaline
PG: prediction group
ROC: receiver operating characteristics
GOT: glutamic oxaloacetic transaminase
GPT: glutamate-pyruvate transaminase
GLDH: glutamate dehydrogenase
MMP: matrix metalloproteinase
